# A shorter distal resection margin is a surrogate marker of nodal metastasis and poor prognosis in distal gastrectomy for advanced gastric cancer

**DOI:** 10.1186/s12885-023-11570-2

**Published:** 2023-11-07

**Authors:** Yusuke Takashima, Shuhei Komatsu, Keiji Nishibeppu, Takuma Ohashi, Toshiyuki Kosuga, Hirotaka Konishi, Atsushi Shiozaki, Takeshi Kubota, Hitoshi Fujiwara, Eigo Otsuji

**Affiliations:** Division of Digestive Surgery, Department of Surgery, Kyoto Prefectural University of Medicine, 465 Kajii-Cho, Kawaramachihirokoji, Kamigyo-Ku, Kyoto, 602-8566 Japan

**Keywords:** Distal surgical margin, Recurrence-free survival, Lymph node metastasis, Gastric cancer

## Abstract

**Background:**

Although a 3–5 cm surgical margin distance is recommended for advanced gastric cancer (GC) in Japanese guidelines, little is known about the clinical effects of the surgical margin, especially the distal resection margin (DM). This study aims to clarify the clinical significance of DM in GC.

**Methods:**

A total of 415 GC patients who underwent curative distal gastrectomy between 2008 and 2018 were analyzed retrospectively.

**Results:**

The DM significantly stratified recurrence-free survival (*P* = 0.002), and a DM < 30 mm was an independent factor of a poor prognosis (*P* = 0.023, hazard ratio: 1.91). Lymphatic recurrence occurred significantly more frequently in the DM < 30 mm group than in the DM ≥ 30 mm group (*P* = 0.019, 6.9% vs. 1.9%). Regarding the station No.6 lymph node metastases in advanced GC (DM < 30 mm vs. 30 mm ≤ DM ≤ 50 mm vs. DM > 50 mm), the number (*P* < 0.001, 1.42 ± 1.69 vs. 1.18 ± 1.80 vs. 0.18 ± 0.64), the positive rate (*P* < 0.001, 59.0% vs. 46.7% vs. 11.3%) and therapeutic value index (43.3 vs. 14.5 vs. 8.0) were significantly higher in the DM < 30 mm group. By subdivision using the DM distance of 30 mm, more segmented prognostic stratifications were possible (*P* < 0.001).

**Conclusions:**

A DM of less than 30 mm could be a surrogate marker of poor RFS, especially increasing nodal recurrence. More intensive treatment strategies, including lymphadenectomy and chemotherapy, are needed for patients with this condition.

**Supplementary Information:**

The online version contains supplementary material available at 10.1186/s12885-023-11570-2.

## Background

Gastric cancer (GC) is one of the most common cancer types and a leading cause of death worldwide [1]. Although various treatments for GC have improved considerably in recent decades [2–4], curative gastrectomy with regional lymph node (LN) dissection remains the primary treatment for patients with resectable GC [5–7]. The surgical procedure recommends a surgical margin of sufficient distance to achieve no residual cancer of the resection line because microscopic positive resection margins have been associated with a poor prognosis for GC [8–11]. However, in patients with a negative surgical margin, it is unclear whether the distance, especially the distal resection margin (DM), affects cancer progression and prognosis after distal GC. A sufficient surgical margin may indicate a more sufficient lymphadenectomy and contribute to adequate local tumor clearance and curability of surrounding metastatic LNs. Indeed, Mine et al. suggested that a proximal margin of more than 2 cm affects a favorable prognosis in patients with Siewert type II and III adenocarcinomas of the esophagogastric junction [12].

Because cancer cells spread stepwise from the peri-gastric area [13], a longer surgical margin may indicate sufficient local tumor clearance to prevent LN metastases (LNM) and perinodal involvement of cancer cells[14]. Regarding the proximal margin, various previous studies have indicated the prognostic effect of a sufficient proximal margin in GC [15–18]. However, there have been no reports of the surgical curability of surrounding LNs and the prognostic effect of DM distance. In the present study, we investigated these issues. Our results suggested that DM distance might be a surrogate marker of local cancer progression and prognosis and could be an indicator of intensive treatments for GC patients.

## Methods

### Patients

**Fig. 1:**
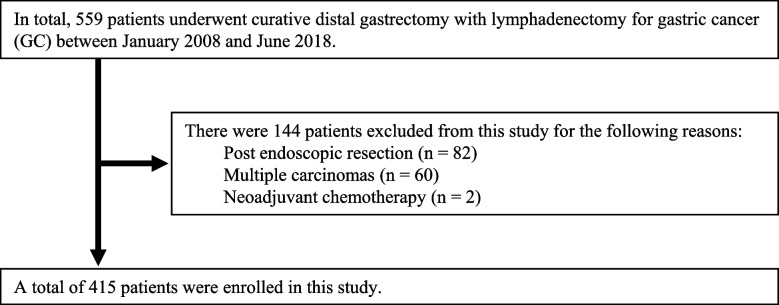
Enrolled patients. A total of 559 patients underwent curative distal gastrectomy with lymphadenectomy between January 2008 and June 2018. Of these, 144 patients were excluded from this study. Thus, data from 415 patients were obtained from their hospital records and retrospectively analyzed

This study was approved by the Kyoto Prefectural University of Medicine and was performed under the ethical standards described in the Declaration of Helsinki. Informed written consent was obtained from all patients. A total of 559 consecutive patients underwent R0 curative distal gastrectomy with lymphadenectomy for GC at our institute between January 2008 and June 2018. Of these, 144 were excluded from this study due to having multiple carcinomas (*n* = 60), post-endoscopic resection (*n* = 82), or neoadjuvant chemotherapy (*n* = 2). Therefore, we investigated 415 consecutive patient**s** (Fig. 1). The follow-up program after gastrectomy consisted of regular physical examinations and laboratory tests, chest X-rays, an upper gastrointestinal series or endoscopy, and ultrasonography or computer tomography for the first 5 years and yearly endoscopies thereafter, if possible. The clinicopathological findings of these patients were retrospectively obtained based on their medical records.

### Definition of the surgical margin

Resected specimens were examined by pathologists and evaluated based on the 15^th^ Japanese Classification of Gastric Carcinoma (JCGC) [19]. All dissected specimens, including the stomach and LNs, were fixed in buffered formalin, embedded in paraffin, and subjected to pathological examination. Pathologists in our institution examined embedded LNs by sectioning slices in the plane of the largest node dimension to confirm the presence of metastasis. The DM distance was defined as the shortest distance from the most distal tumor end to the distal resection line, measured on formalin-fixed surgical specimens. We defined pT2 or deeper, and/or pN1 or higher as advanced GC, and the remaining pT1 cancers as early GC.

### Statistical analysis

Statistical analyses were conducted using JMP version 16 (SAS Institute Inc., Cary, NC, USA). Mann–Whitney U tests for unpaired continuous data were used to compare clinicopathological variables. For survival analysis, Kaplan–Meier survival curves were constructed for groups based on univariate predictors, and differences between the groups were tested using generalized Wilcoxon tests. A Cox proportional hazards model was used to further evaluate the multivariate survival analysis. Statistical significance was set at *P* < 0.05.

## Results

### Shorter DM distance showed poor prognosis in patients who received curative distal gastrectomy

**Fig. 2:**
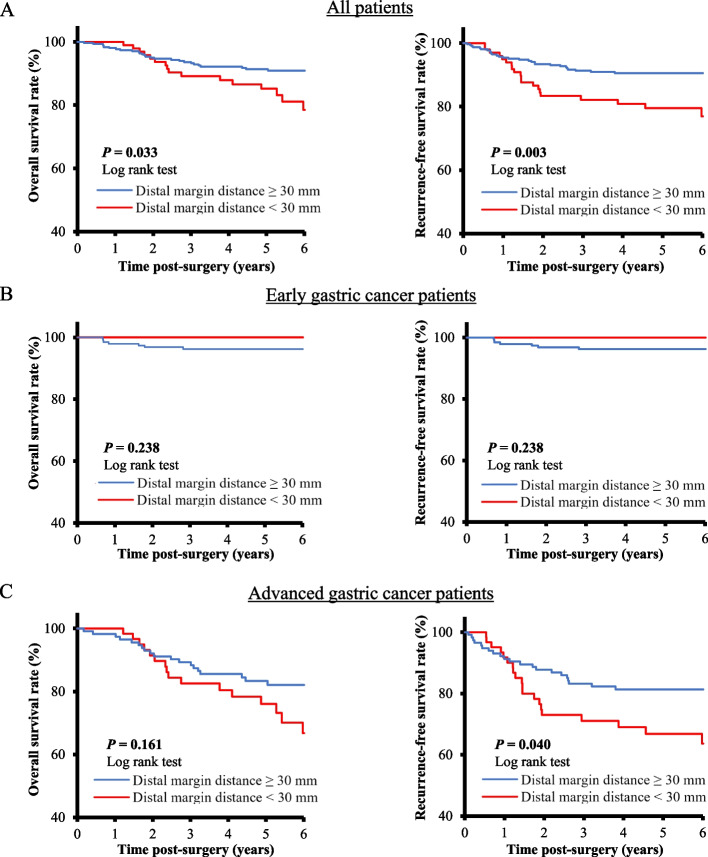
Comparisons of survival curves according to the distal resection margin (DM) distance. **A** Comparisons of overall survival (OS) and recurrence-free survival (RFS) in gastric cancer patients who underwent distal gastrectomy. **B** Comparisons of OS and RFS in early gastric cancer patients who underwent distal gastrectomy. **C** Comparisons of OS and RFS in advanced gastric cancer patients who underwent distal gastrectomy

Firstly, we investigated differences in the prognosis of patients who received curative distal gastrectomy according to the DM distance using cut-off values of 30 mm and 50 mm, which are recommended as surgical margins for localized GC (Type 1 or 2) and diffuse GC (Type 3 or 4) in the Japanese guidelines. As a result, the DM distance significantly stratified recurrence-free survival (RFS), and the DM distance < 30 mm group showed the worst RFS rate (DM distance < 30 mm vs. 30 mm ≤ DM distance ≤ 50 mm vs. DM distance > 50 mm: 77.0% vs. 84.2% vs. 92.8%, *P* = 0.002; Figure S1). Next, we investigated the prognosis of GC patients separately according to the degree of cancer progression using a cut-off value of 30 mm (Fig. 2). In advanced GC, patients with a DM distance < 30 mm had significantly poorer RFS than those with a DM distance ≥ 30 mm (DM distance < 30 mm vs. DM distance ≥ 30 mm: 63.7% vs. 80.6%, *P* = 0.040), while there was no significant difference between both groups in early GC (RFS: DM distance < 30 mm vs. DM distance ≥ 30 mm: 100% vs. 96.2%, *P* = 0.238).

### Univariate and multivariate analyses of DM distance using a Cox proportional hazard model

To elucidate the prognostic factors of RFS in patients who underwent distal gastrectomy, we performed univariate and multivariate analyses using a Cox proportional hazard model. As shown in Table 1, the clinical variables included pStage, histological type, venous invasion, lymphatic invasion, adjuvant chemotherapy, tumor axis, and distal surgical margin distance. The median tumor axis in this cohort was 35 mm (Interquartile range, 23–53), therefore, we used 35 mm as the cut-off. Consequently, univariate and multivariate analyses showed that a DM distance < 30 mm was an independent poor prognostic factor for RFS (*P* = 0.023, hazard ratio [HR]: 1.91, 95% confidence interval [CI]: 1.09–3.33) as well as a factor of advanced pStage.

**Table 1:** Results of univariate and multivariate analyses using a Cox proportional hazard model

Variables	Univariate^a^	Multivariate^b^		
	*P*-value	HR^c^	95% CI^d^	*P-*value
pStage				
II / III *vs*. I	**< 0.001**	4.78	2.24–10.20	**< 0.001**
Histological type				
Undifferentiated *vs*. differentiated	0.817	1.07	0.61–1.87	0.808
Venous invasion				
Positive *vs*. negative	**< 0.001**	1.24	0.67–2.32	0.496
Lymphatic invasion				
Positive *vs*. negative	**< 0.001**	1.26	0.66–2.42	0.486
Adjuvant chemotherapy				
Positive vs. negative	**0.033**	0.60	0.32–1.12	0.108
Tumor axis (mm)				
35 ≦ *vs*. < 35	**< 0.001**	1.96	0.93–4.14	0.078
Distal surgical margin (mm)				
< 30 *vs*. 30 ≦	**0.003**	1.91	1.09–3.33	**0.023**

Significant *P*-values are shown in bold

^a^Analyzed by log-rank (Mantel-Cox) test

^b^Analyzed by a Cox proportional hazard model

^c^*HR* hazard ratio

^d^*CI* confidence interval

### Comparisons of clinicopathological factors between the DM distance < 30 mm group and the DM distance ≥ 30 mm group

Next, we compared the clinicopathological characteristics of the DM distance < 30 mm group and the DM distance ≥ 30 mm group (Table 2). There was no difference between the groups in patients with advanced GC, except for the proportion of tumors located in the upper stomach (*P* < 0.001) and larger tumor axis (*P* = 0.005). A DM distance < 30 mm was significantly correlated with the differentiated type (*P* = 0.023) and a higher rate of positive lymphatic invasion (*P* = 0.036) compared to patients with early GC with a DM distance ≥ 30 mm. Besides, multivariate analysis using the Cox proportional hazard model to assess the prognostic factors of RFS in advanced GC showed that a DM distance < 30 mm, rather than a larger tumor axis, was identified as an independent poor prognostic factor (*P* = 0.019, hazard ratio [HR]: 2.10, 95% confidence interval [CI]: 1.13–3.88; Table S1).

**Table 2:** Comparisons of clinicopathological factors between the distal margin (DM) distance < 30 mm group and the DM distance ≥ 30 mm group

		Early gastric cancer					Advanced gastric cancer					
Variables	n	DM < 30 mm *n* = 40		DM ≥ 30 mm *n* = 198		*P-*value^*^	n	DM < 30 mm *n* = 61		DM ≥ 30 mm *n* = 116		*P-*value^*^
Sex						0.861						0.410
Male	136	22	(55.0%)	114	(57.6%)		115	37	(60.7%)	78	(67.2%)	
Female	102	18	(45.0%)	84	(42.4%)		62	24	(39.3%)	38	(32.8%)	
BMI (kg/m^2^)						0.139						0.433
< 25	188	28	(70.0%)	160	(80.8%)		141	51	(83.6%)	90	(77.6%)	
≥ 25	50	12	(30.0%)	38	(19.2%)		36	10	(16.4%)	26	(22.4%)	
Age (years)						0.864						0.333
< 65	117	19	(47.5%)	98	(49.5%)		110	20	(32.8%)	47	(40.5%)	
≥ 65	121	21	(52.5%)	100	(50.5%)		67	41	(67.2%)	69	(59.5%)	
Location						**< 0.001**						** < 0.001**
U	6	0	(0%)	6	(3.0%)		2	0	(0%)	2	(1.8%)	
M	153	8	(23.7%)	145	(73.3%)		81	7	(12.7%)	74	(64.9%)	
L	69	32	(73.2%)	47	(23.7%)		86	48	(87.3%)	38	(33.3%)	
Tumor axis (mm)						0.859						**0.005**
< 35	147	24	(60.0%)	123	(62.1%)		42	7	(11.5%)	35	(30.2%)	
≥ 35	91	16	(40.0%)	75	(37.9%)		135	54	(88.5%)	81	(69.8%)	
pT-stage						0.298						0.974
T1	238	40		198			30	11		19		
1a (m)	132	19	(47.5%)	113	(57.1%)		1	0	(0%)	1	(0.9%)	
1b (sm)	106	21	(52.5%)	85	(42.9%)		29	11	(18.0%)	18	(15.5%)	
T2	0	0	(0%)	0	(0%)		53	17	(27.9%)	36	(31.0%)	
T3	0	0	(0%)	0	(0%)		65	23	(37.7%)	42	(36.2%)	
T4	0	0	(0%)	0	(0%)		29	10	(16.4%)	19	(16.4%)	
pN-stage						1.000						0.525
N0	238	40	(100%)	198	(100%)		61	17	(27.8%)	44	(38.0%)	
N1	0	0	(0%)	0	(0%)		44	16	(26.2%)	28	(24.0%)	
N2	0	0	(0%)	0	(0%)		38	16	(26.2%)	22	(19.0%)	
N3	0	0	(0%)	0	(0%)		34	12	(19.7%)	22	(19.0%)	
Histopathological type						**0.023**						1.000
Differentiated	108	25	(62.5%)	83	(41.9%)		80	28	(45.9%)	52	(44.8%)	
Undifferentiated	130	15	(37.5%)	115	(58.1%)		96	33	(54.1%)	64	(55.2%)	
Venous invasion						0.417						0.554
Absent	211	34	(85.0%)	177	(89.4%)		73	27	(44.3%)	46	(39.7%)	
Present	27	6	(15.0%)	21	(10.6%)		104	34	(55.7%)	70	(60.3%)	
Lymphatic invasion						**0.036**						0.505
Absent	197	28	(70.0%)	169	(85.3%)		49	15	(24.6%)	34	(29.3%)	
Present	41	12	(30.0%)	29	(14.7%)		128	46	(75.4%)	82	(70.7%)	
Adjuvant chemotherapy						1.000						0.491
Absent	238	40	(100%)	198	(100%)		102	33	(54.1%)	69	(59.5%)	
Present	0	0	(0%)	0	(0%)		75	28	(45.9%)	47	(40.5%)	
Lymphadenectomy extent						0.495						0.524
≤ D1	10	2	(5.0%)	8	(3.5%)		140	50	(82.0%)	90	(77.6%)	
D1 +	218	35	(87.5%)	183	(92.4%)		35	11	(18.0%)	24	(20.7%)	
D2≦	10	3	(7.5%)	7	(4.1%)		2	0	(0%)	2	(1.7%)	

Significant *P*-values are shown in bold

^*^P-values are from the chi-squared test or Fisher’s exact probability test

### Contribution of a shorter DM distance to station No.6 LNM

**Fig. 3:**
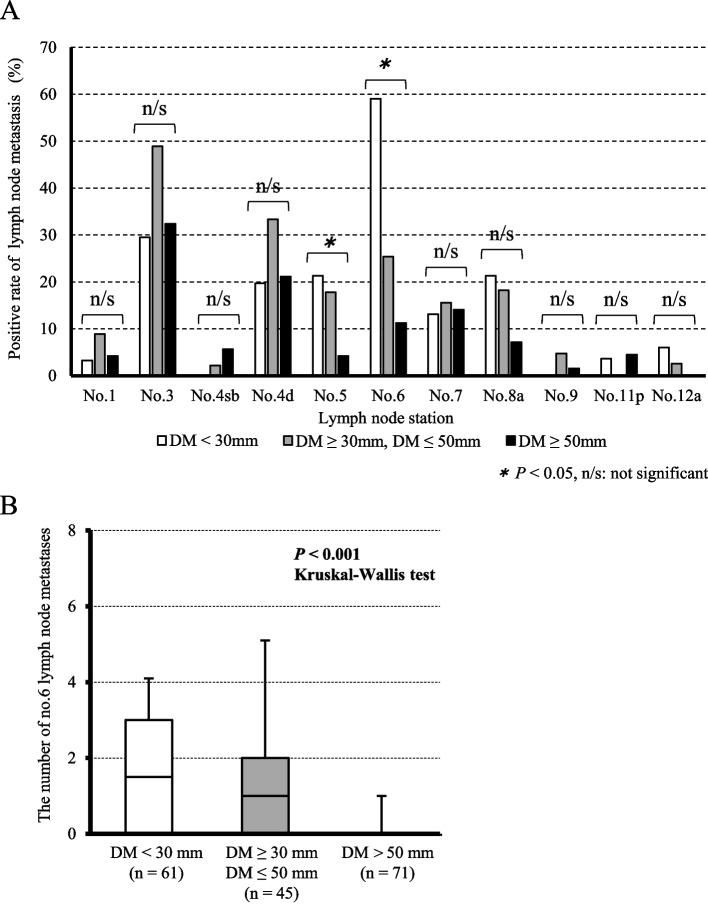
Comparisons of the incidence of lymph node metastasis between advanced gastric cancer (GC) patients with a distal resection margin (DM) distance ≥ 30 mm and a DM distance < 30 mm***.* A** In advanced GC, there was a higher incidence of lymph node metastasis at station No.6 in the DM distance < 30 mm group compared to the DM distance ≥ 30 mm. **B** The number of lymph node metastases at station No.6 was higher in the DM distance < 30 mm group compared to the DM distance ≥ 30 mm and the DM distance ≤ 50 mm group, and the DM > 50 mm group

Regarding the patterns of recurrence, the incidence of lymphatic recurrence was significantly higher in patients with a DM distance < 30 mm compared to those with a DM distance ≥ 30 mm (DM distance < 30 mm vs. DM distance ≥ 30 mm: 6.9% vs. 1.9%, *P* = 0.019; Table 3). Among seven cases of lymphatic recurrence in the DM distance < 30 mm group, six cases occurred in the para-aortic LN station and the hepatoduodenal ligament LN station. To investigate the mechanism by which a shorter DM distance is associated with a higher frequency of LN recurrence, we compared the frequency of the LNMs in each station. In the patients with advanced GC, the LNMs in station No.6 occurred significantly more frequently (DM distance < 30 mm vs. 30 mm ≤ DM distance ≤ 50 mm vs. DM distance > 50 mm: 59.0% vs. 46.7% vs. 11.3%, *P* < 0.001) and the number of LNMs in station No.6 was significantly higher (DM distance < 30 mm vs. 30 mm ≤ DM distance ≤ 50 mm vs. DM distance > 50 mm: 1.42 ± 1.69 vs. 1.18 ± 1.80 vs. 0.18 ± 0.64, *P* < 0.001) in the DM distance < 30 mm group than in the DM distance ≥ 30 mm group (Fig. 3). To determine more accurately whether the ease of metastasis to the No.6 lymph node is dependent on the DM distance, we performed subgroup analysis among the patients with pN1. This analysis showed that LNM in station No.6 occurred more frequently in the DM distance < 30 mm group than in the other groups (DM distance < 30 mm vs. 30 mm ≤ DM distance ≤ 50 mm vs. DM distance > 50 mm: 75.0% vs. 45.5% vs. 13.3%, *P* = 0.003; Table S2). In addition, the therapeutic value index was estimated by multiplying the metastasis rate by the 5-year overall survival rate in patients with metastasis in the respective nodes [20]. As a result, the therapeutic value index in station No.6 LNs was highest in the DM distance < 30 mm group than in the other two groups in advanced GC (DM distance < 30 mm vs. 30 mm ≤ DM distance ≤ 50 mm vs. DM distance > 50 mm: 43.3 vs. 14.5 vs. 8.0; Table 4).

**Table 3:** Associations between the distal margin and disease recurrence in GC patients with gastrectomy

	All patients			
	n	Distal margin		*P-*value^*^
		< 30 mm	≥ 30 mm	
Number of patients	415	101	314	
Total recurrences	35	18 (17.8%)	17 (5.4%)	**< 0.001**
Hematogenous recurrences	9	3 (3.0%)	6 (1.9%)	0.460
Lymphatic recurrences	13	7 (6.9%)	6 (1.9%)	**0.019**
Peritoneal recurrences	10	5 (5.0%)	5 (1.6%)	0.068
Local recurrences	2	2 (2.0%)	0 (0%)	0.059

Significant values are shown in bold

^*^P-values are from Fischer’s exact test

**Table 4:** Therapeutic value of lymphadenectomy in respective lymph nodes in advanced gastric cancer

Number of lymph nodes	Metastatic rate (%)	5 yr-OS (%)	Therapeutic value index (%)
No.1			
DM < 30 mm	3.3	0	0
DM ≥ 30 mm, DM ≤ 50 mm	8.9	50.0	4.4
DM > 50 mm	4.2	100	4.2
No.3			
DM < 30 mm	29.5	61.9	18.3
DM ≥ 30 mm, DM ≤ 50 mm	48.9	65.8	32.2
DM > 50 mm	32.4	80.7	26.1
No.4sb			
DM < 30 mm	0	-	-
DM ≥ 30 mm, DM ≤ 50 mm	2.2	37.5	0.8
DM > 50 mm	5.6	0	0
No.4d			
DM < 30 mm	19.7	57.8	11.4
DM ≥ 30 mm, DM ≤ 50 mm	33.3	50.0	16.7
DM > 50 mm	21.1	83.6	17.7
No.5			
DM < 30 mm	21.3	59.8	12.7
DM ≥ 30 mm, DM ≤ 50 mm	17.8	37.5	6.7
DM > 50 mm	4.2	100	4.2
No.6			
DM < 30 mm	59.0	73.4	43.3
DM ≥ 30 mm, DM ≤ 50 mm	25.4	57.1	14.5
DM > 50 mm	11.3	71.4	8.0
No.7			
DM < 30 mm	13.1	41.7	5.5
DM ≥ 30 mm, DM ≤ 50 mm	15.6	66.7	10.4
DM > 50 mm	14.1	100	14.1
No.8a			
DM < 30 mm	21.3	68.4	14.6
DM ≥ 30 mm, DM ≤ 50 mm	18.2	37.5	6.8
DM > 50 mm	7.1	100	7.1
No.9			
DM < 30 mm	0	-	-
DM ≥ 30 mm, DM ≤ 50 mm	4.8	0	0.0
DM > 50 mm	1.6	100	1.6
No.11p			
DM < 30 mm	3.6	100	3.6
DM ≥ 30 mm, DM ≤ 50 mm	0	-	-
DM > 50 mm	4.5	100	4.5
No.12a			
DM < 30 mm	6.0	66.7	4.0
DM ≥ 30 mm, DM ≤ 50 mm	2.6	0	0
DM > 50 mm	0	-	-

The therapeutic value index was calculated by multiplying the metastatic rate by the 5-year overall survival rate in patients with metastasis in the respective nodes

### Evaluation of the more precise staging system using a 30 mm cut-off value for the DM distance

**Fig. 4:**
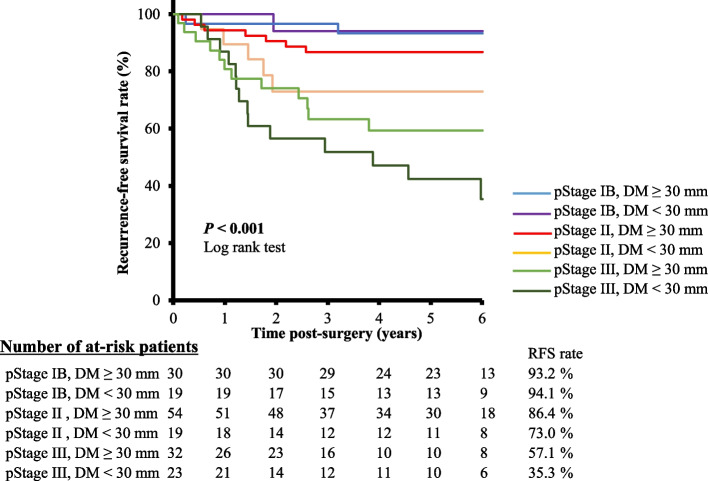
Recurrence-free survival curves with combinations of the staging system of the Japanese classification of gastric carcinoma (JCGC) and the distal resection margin (DM) distance. Greater segmented prognostic stratification was possible with combinations of the staging system in JCGC and using a DM distance cut-off value of 30 mm (RFS; pStage IB, DM < 30 mm vs. pStage IB, DM ≥ 30 mm, vs. pStage II, DM < 30 mm vs. pStage II, DM ≥ 30 mm vs. pStage III, DM < 30 mm vs. pStage III, DM ≥ 30 mm = 94.1% vs. 93.2% vs. 73.0% vs. 86.4% vs. 35.3% vs. 57.1%, *P* < 0.001)

Next, we examined survival curves for combinations of the staging system in JCGC with a DM distance of 30 mm. More segmented prognostic stratifications were observed (RFS; pStage II, DM distance < 30 mm vs. pStage II, DM distance ≥ 30 mm vs. pStage III, DM distance < 30 mm vs. pStage III, DM distance ≥ 30 mm: 86.4% vs. 73.0% vs. 57.1% vs. 35.3%, *P* < 0.001; Fig. 4). This result suggested that a cut-off value for the DM distance of < 30 mm could be a useful indicator for stratifying prognosis as well as the traditional staging system in patients with pStage II or III GC.

## Discussion

The Japanese Gastric Cancer Association (JGCA) recommends a margin of 2 cm for T1 tumors, 3 cm for T2 or deeper tumors with an expansive growth pattern, and 5 cm for T2 or deeper tumors with an infiltrative growth pattern [6]. On the other hand, the European Society for Medical Oncology indicates an appropriate proximal surgical margin of 5–8 cm [5], and The National Comprehensive Cancer Network guidelines suggest an adequate surgical margin of 4 cm for T1b-T3 tumors to achieve negative microscopic margin [21]. Although some clinical studies have investigated the prognostic significance of proximal surgical margin distance [17, 18, 22–26], to our knowledge, there have been no reports of the prognostic and treatment significance of the DM distance. In this study, we first investigate the prognostic effect of the DM distance using the cut-off of 3 cm and 5 cm as cut-off values based on Japanese guidelines and previous studies on proximal margin distances [18, 25] and found that the patients with a DM distance of less than 3 cm had a significantly worse prognosis. Therefore, we selected 3 cm as the cut-off value for further analyses. Then, we clearly demonstrated that a DM distance < 30 mm was an independent factor of a poor prognosis (HR: 1.91, 95% CI: 1.09–3.33) and an indicator of excessive nodal metastases around the No.6 LN station. These findings strongly suggested that DM distance could be an important surrogate marker for nodal metastasis and poor prognosis, applying more intensive lymphadenectomy and adjuvant therapy for advanced GC patients.

Previously, Maspero et al. retrospectively investigated the adequacy of the resection margin in 279 stage I – III GC patients and proved that adequate margins contributed to favorable OS, RFS, and local recurrence rates [26]. Another two reports also demonstrated that a longer proximal surgical margin distance contributed to long-term prognosis for GC [17, 24]. However, Kim et al. demonstrated that proximal margin distance did not affect OS and RFS in multivariate analysis using a Cox proportion hazard model in a large cohort of 859 cases of distal gastrectomy and 659 cases of total gastrectomy, and another two studies also stated similar results [22, 23]. Although one study found that the proximal margin distance contributed to curability [15], most studies do not support this outcome. Therefore, the curative and prognostic effects of proximal margin distance are still controversial. Regarding the distal resection margin, little is known about its clinical effects. In this study, we clearly demonstrated that a shorter DM distance contributed to lymphatic spreading as well as a poorer prognosis.

The most striking finding in the present study was that lymphatic recurrence occurred more frequently in patients with a DM distance < 30 mm compared to a DM ≥ 30 mm, and a positive rate of LNMs at station No.6 (DM distance < 30 mm vs. 30 mm ≤ DM distance ≤ 50 mm vs. DM distance > 50 mm: 59.0% vs. 46.7% vs. 11.3%, *P* < 0.001) in advanced GC, which might indicate a putative additional lymphatic flow to station No.14v and No.16 LNs outside the LN resection area [6, 13], and the number of LNMs at station No.6 (*P* < 0.001: DM distance < 30 mm vs. 30 mm ≤ DM distance ≤ 50 mm vs. DM distance > 50 mm: 1.42 ± 1.69 vs. 1.18 ± 1.80 vs. 0.18 ± 0.64) was significantly higher in the DM distance < 30 mm group than in the DM distance ≥ 30 mm group. Thus, DM distance could be considered an indicator of the extent of lymphatic spreading in GC. In distal gastrectomy, unlike the proximal resection margin, the distal resection margin is subject to anatomical constraints that allow for relatively little adjustment. As a result, the distance of the distal margin can be considered an indicator of the degree of distal localization of the tumor. Indeed, in adenocarcinoma of the esophagogastric junction (EGJ), previous studies have investigated the relationship between mediastinal LNMs and the extent of esophageal invasion in adenocarcinoma and found that a longer distance from the EGJ to the proximal edge of the primary tumor contribute to middle or upper mediastinal LNs [27–29]. These findings strongly suggested the significance of the DM distance on the extent and pattern of nodal metastasis.

Regarding the therapeutic value of lymphadenectomy associated with the DM distance, the therapeutic value index of LNs at station No.6 was extremely higher in GC patients with a DM distance < 30 mm than in those with a DM distance ≥ 30 mm. This finding also indicated that GC patients with a DM distance < 30 mm may need additional lymphadenectomy around LNs at station No.6. Regarding D2 + lymphadenectomy, recent studies have clarified the significance of station No.13, 14v, and 16 LN dissection [30–33] in some advanced types of GC. Moreover, some studies have identified that LNM at station No.6 was an independent risk factor for station No.13 and 14v LNM [30, 31]. In our study, we demonstrated that the DM distance was a surrogate marker of the LNM pattern and served as an indicator to reconsider the extent of LN dissection.

It is crucial to note that our proposal is not advocating for the enlargement of the surgical resection margin; rather, it suggests a reevaluation of more suitable strategies, considering tumor laterality using such as the indicator of DM distance. In distal gastrectomy, anatomical constraints may make extending the surgical resection margin challenging in some instances. Consequently, in cases of distal gastric cancer where obtaining a sufficient DM distance is not feasible, it becomes necessary to consider broader lymph node dissection beyond the standard scope and/or more potent adjuvant chemotherapy methods, such as Cape-OX therapy, SOX therapy, and S-1 plus docetaxel therapy [34–36].

This study had some limitations. Firstly, because the results were obtained from a retrospective evaluation of a small number of patients at a single institute, the findings of the present study should be validated in a larger prospective multicenter study. Secondly, the DM distance was used in this study as an indicator of distal orientation; however, the actual duodenal resection length varies and needs to be validated in more detail. Thirdly, there was the potential variability in the measurement of margin distances due to different formalin immersion times, which can cause changes in tissue shrinkage. Fourthly, there was a discrepancy between RFS and OS among patients with advanced GC. The reason for this could be that of the 34 patients who experienced recurrence events, 4 (11.8%) have survived for more than 5 years due to the improvement and efficacy of recent chemotherapies for recurrences. It is also important to note that further research is needed, particularly with larger sample sizes and longer follow-up periods, to corroborate these findings. Nevertheless, our findings suggest the impact of DM distance on cancer progression and prognosis for GC patients due to local recurrence and lymphatic progression. Moreover, we should discuss that patients with a shorter DM distance could be sufficiently treated and obtain benefits from more extended lymphadenectomy and/or more intensive adjuvant therapy due to the higher incidence of LN involvement.

## Conclusion

A shorter DM distance could be an indicator of poor prognosis in patients with advanced GC who underwent distal gastrectomy, increasing the frequency of metastasis to LNs at station No.6. Further studies are warranted to clarify the significance of additional extended lymphadenectomy around station No.6 LNs, such as station No.14v, and intensive adjuvant chemotherapy to improve the prognosis of patients with a shorter DM distance.

### Supplementary Information

**Additional file 1: Figure S1.** Comparisons of recurrence-free survival curves according to the distal resection margin distance.

**Additional file 2: Table S1.** Results of univariate and multivariate analyses using a Cox proportional hazard model in advanced gastric cancer. **Table S2.** Frequency of No.6 lymph node metastasis in patients with pN1.

## Data Availability

The datasets used during the current study are available from the corresponding author on reasonable request.

## Abbreviation

GC: Gastric cancer
RFS: Recurrence-free survival
DM: Distal margin
LN: Lymph node
JCGC: Japanese Classification of Gastric Carcinoma
