# Supplementary material for: A shorter distal resection margin is a surrogate marker of nodal metastasis and poor prognosis in distal gastrectomy for advanced gastric cancer
Source: BMC Cancer. 2023 Nov 7;23:1075. doi: 10.1186/s12885-023-11570-2 (PMC10629168; doi:10.1186/s12885-023-11570-2)
Supplement: Supplementary file 1 — Additional file 1: Figure S1. Comparisons of recurrence-free survival curves according to the distal resection margin distance. [file 12885_2023_11570_MOESM1_ESM.docx]

**Figure S1**: Comparisons of recurrence-free survival curves according to the distal resection margin distance


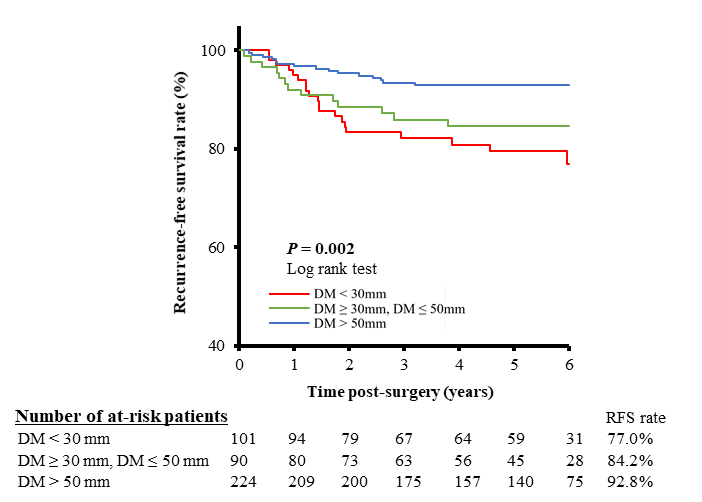


The DM distance significantly stratified recurrence-free survival (DM distance < 30 mm vs. 30 mm ≤ DM distance ≤ 50 mm vs. DM distance > 50 mm: 77.0% vs. 84.2% vs. 92.8%, *P* = 0.002).
