# Supplementary material for: A shorter distal resection margin is a surrogate marker of nodal metastasis and poor prognosis in distal gastrectomy for advanced gastric cancer
Source: BMC Cancer. 2023 Nov 7;23:1075. doi: 10.1186/s12885-023-11570-2 (PMC10629168; doi:10.1186/s12885-023-11570-2)
Supplement: Supplementary file 2 — Additional file 2: Table S1. Results of univariate and multivariate analyses using a Cox proportional hazard model in advanced gastric cancer. Table S2. Frequency of No.6 lymph node metastasis in patients with pN1. [file 12885_2023_11570_MOESM2_ESM.docx]

**Table S1**: Results of univariate and multivariate analyses using a Cox proportional hazard model in advanced gastric cancer

| Variables | | Univariate^a^ |  | Multivariate^b^ | | | |
| --- | --- | --- | --- | --- | --- | --- | --- |
|  |  | *P*-value |  | HR^c^ | 95% CI^d^ |  | *P-*value |
| pStage | |  |  |  |  |  |  |
|  | II / III *vs*. I | **0.001** |  | 3.83 | 1.45-10.11 |  | **0.007** |
| Histological type | |  |  |  |  |  |  |
|  | Undifferentiated *vs*. differentiated | 0.410 |  | 0.79 | 0.43-1.47 |  | 0.465 |
| Venous invasion | |  |  |  |  |  |  |
|  | Positive *vs*. negative | 0.422 |  | 1.05 | 0.54-2.03 |  | 0.882 |
| Lymphatic invasion | |  |  |  |  |  |  |
|  | Positive *vs*. negative | 0.202 |  | 1.08 | 0.53-2.19 |  | 0.840 |
| Adjuvant chemotherapy | |  |  |  |  |  |  |
|  | Positive vs. negative | 0.402 |  | 0.58 | 0.31-1.09 |  | 0.091 |
| Tumor axis (mm) | |  |  |  |  |  |  |
|  | 35 ≦ *vs*. < 35 | **0.012** |  | 2.39 | 0.82-7.01 |  | 0.111 |
| Distal surgical margin (mm) | |  |  |  |  |  |  |
|  | < 30 *vs*. 30 ≦ | **0.031** |  | 2.10 | 1.13-3.88 |  | **0.019** |

^a^Analyzed by log-rank (Mantel-Cox) test; ^b^Analyzed by a Cox proportional hazard model; ^c^HR: hazard ratio; ^d^CI: confidence interval.

Significant *P*-values are shown in bold.

**Table S2**: Frequency of No.6 lymph node metastasis in patients with pN1

| Distal surgical margin distance | | Total | Number of patients with No.6 lymph node metastasis | *P-*value^*^ |
| --- | --- | --- | --- | --- |
| < 30 mm |  | 16 | 12 (75.0%) | **0.003** |
| ≥ 30 mm and ≤ 50 mm |  | 13 | 6 (45.5%) |  |
| ≧ 50 mm |  | 15 | 2 (13.3%) |  |

^*^*P*-values are from the chi-squared test.

Significant *P*-values are shown in bold.
